# Measurement of tibial nerve excursion during ankle joint dorsiflexion in a weight-bearing position with ultrasound imaging

**DOI:** 10.1186/1757-1146-5-5

**Published:** 2012-03-08

**Authors:** Matthew Carroll, Janet Yau, Keith Rome, Wayne Hing

**Affiliations:** 1 School of Rehabilitation & Occupation Studies, Health & Rehabilitation Research Institute, Department of Podiatry, AUT University, Private Bag 92006, Auckland 1142, New Zealand; 2 School of Rehabilitation & Occupation Studies, Health & Rehabilitation Research Institute, AUT University, Private Bag 92006, Auckland 1142, New Zealand

## Abstract

**Background:**

The ability of peripheral nerves to stretch and slide is thought to be of paramount importance to maintain ideal neural function. Excursion in peripheral nerves such as the tibial can be measured by analysis of ultrasound images. The aim of this study was to assess the degree of longitudinal tibial nerve excursion as the ankle moved from plantar flexion to dorsiflexion in a standardised weight-bearing position. The reliability of ultrasound imaging to measure tibial nerve excursion was also quantified.

**Methods:**

The tibial nerve was imaged over two separate sessions in sixteen asymptomatic participants in a weight-bearing position. Longitudinal nerve excursion was calculated from a three-second video loop captured by ultrasound imaging using frame-by-frame cross-correlation analysis. Intraclass correlation coefficients (ICC) with 95% confidence intervals (CI) were used to assess the intra-rater reliability. Standard error of the measurement (SEM) and smallest real difference (SRD) were calculated to assess measurement error.

**Results:**

Mean nerve excursion was 2.99 mm SEM ± 0.22 mm. The SRD was 0.84 mm for session 1 and 0.66 mm for session 2. Intra-rater reliability was excellent with an ICC = 0.93.

**Conclusions:**

Assessment of real-time ultrasound images of the tibial nerve via frame-by-frame cross-correlation analysis is a reliable non-invasive technique to assess longitudinal nerve excursion. The relationship between foot posture and nerve excursion can be further investigated.

## Introduction

During the gait cycle lower extremity motions such as ankle joint dorsiflexion and pronation of the foot will require the tibial nerve to adapt to positional change imposed by joint motions. To accommodate for positional joint change the tibial nerve possesses mechanical properties which enable it to withstand compression, adapt to repetitive force and stretch and slide in relation to the surrounding tissues [1]. The ability of peripheral nerves to stretch and slide is thought to be of paramount importance to maintain ideal neural function [1-3].

Peripheral nerve compression may disrupt the ability of the nerve to stretch and slide [4]. Prolonged compression creates a sequelae of intraneural events that may ultimately lead to impaired nerve sliding [4]. Although compression may affect the mechanical functioning of the tibial nerve, no studies have quantified the degree of *in-vivo *longitudinal tibial nerve excursion that can be considered normal in a non-pathological state. *In-vitro *methodologies have demonstrated that 5% - 10% elongation results in impaired blood flow [5,6], with complete intraneural circulation at approximately 15% elongation [7]. Cadervic-based studies have demonstrated tibial nerve movement of 9.5 mm [8] and 6.9 mm [9] as the ankle was moved into dorsiflexion.

Real-time ultrasound imaging provides a non-invasive means of measuring *in-vivo *longitudinal nerve excursion. Advances in ultrasound imaging equipment and the development of specific frame-by-frame cross-correlation analysis software have made it possible to analyse real-time ultrasound images, allowing for quantification of *in-vivo *peripheral nerve excursion [10]. *In-vivo *investigation of nerve motion through ultrasound imaging and cross-correlation analysis has predominately focused on the upper limb, particularly the median and ulnar nerve [11-20] and to the authors knowledge only one study has used ultrasound imaging and cross-correlation analysis to determine nerve excursion in the lower limb, focusing on the sciatic nerve [21].

Though cross-correlation analysis has become increasingly used to assess nerve excursion, only three studies have assessed the reliability of the technique [10,20,21]. Dilley et al. [10] used pilot phantom and *in-vivo *controls to assess reliability. The results indicated the cross-correlation method produced similar results between the phantoms and *in-vivo *median nerve excursion, varying only 10% between trials, with a low within-session variability SD: 0.2-0.4 mm. Coppieters et al. [20] assessed the inter-rater reliability through measurement of longitudinal excursion of the median nerve. Results indicated excellent inter-rater reliability ICC = 0.96, 95% CI: 0.883, 0.988; SEM, 0.66 mm. In the only reliability study related to the lower limb, Ellis et al. [21] investigated the intra-rater reliability of diagnostic ultrasound to measure longitudinal sciatic nerve excursion at the posterior midthigh and popliteal fossa. Ultrasound images of longitudinal sciatic nerve excursion demonstrated good intra-rater reliability at the posterior mid-thigh.

To date, there is limited evidence regarding *in-vivo *measurement of tibial nerve excursion. Therefore, the aims of this study were firstly, to quantify the degree of tibial nerve excursion during ankle joint dorsiflexion in a weight-bearing position. The second aim was to assess the intra-rater reliability of measuring longitudinal tibial nerve excursion in a weight-bearing position, using ultrasound imaging and frame-by-frame cross-correlation analysis.

## Methods

### Participants

Sixteen participants (10 female, 6 male) were recruited from a university population with a mean (SD) age of 34.7 years (9.3), mean (SD) weight of 73.6 Kg (15.1), mean (SD) height of 173.4 cm (10.4) and mean (SD) BMI of 24.2 kg/m2 (3.4). All imaging was conducted in a private ultrasongraphy clinic based on campus over 4 week duration. Participants were excluded if they were under twenty years old, had a history of heel pain in the last six months, a previous history of lower limb surgery, foot arthritis, neuropathic disease, neuromuscular disease or if the participant required aids to walk. Ethical approval was granted by the University Ethics Committee. Informed consent was given by all participants prior to testing.

### Equipment

B-mode real time ultrasound and colour imaging was performed using a Philips iU22 ™ ultrasound unit with a linear array transducer (17-5 MHz). Aquasonic 100^® ^ultrasound gel was applied directly onto the participant's skin, superior to the medial malleoli prior to ultrasound scanning. All ultrasound imaging was performed by one examiner [MC]. The examiner underwent 3 months of training under an experienced ultrasonagrapher prior to commencement of this project.

### Procedure

**Figure 1:**
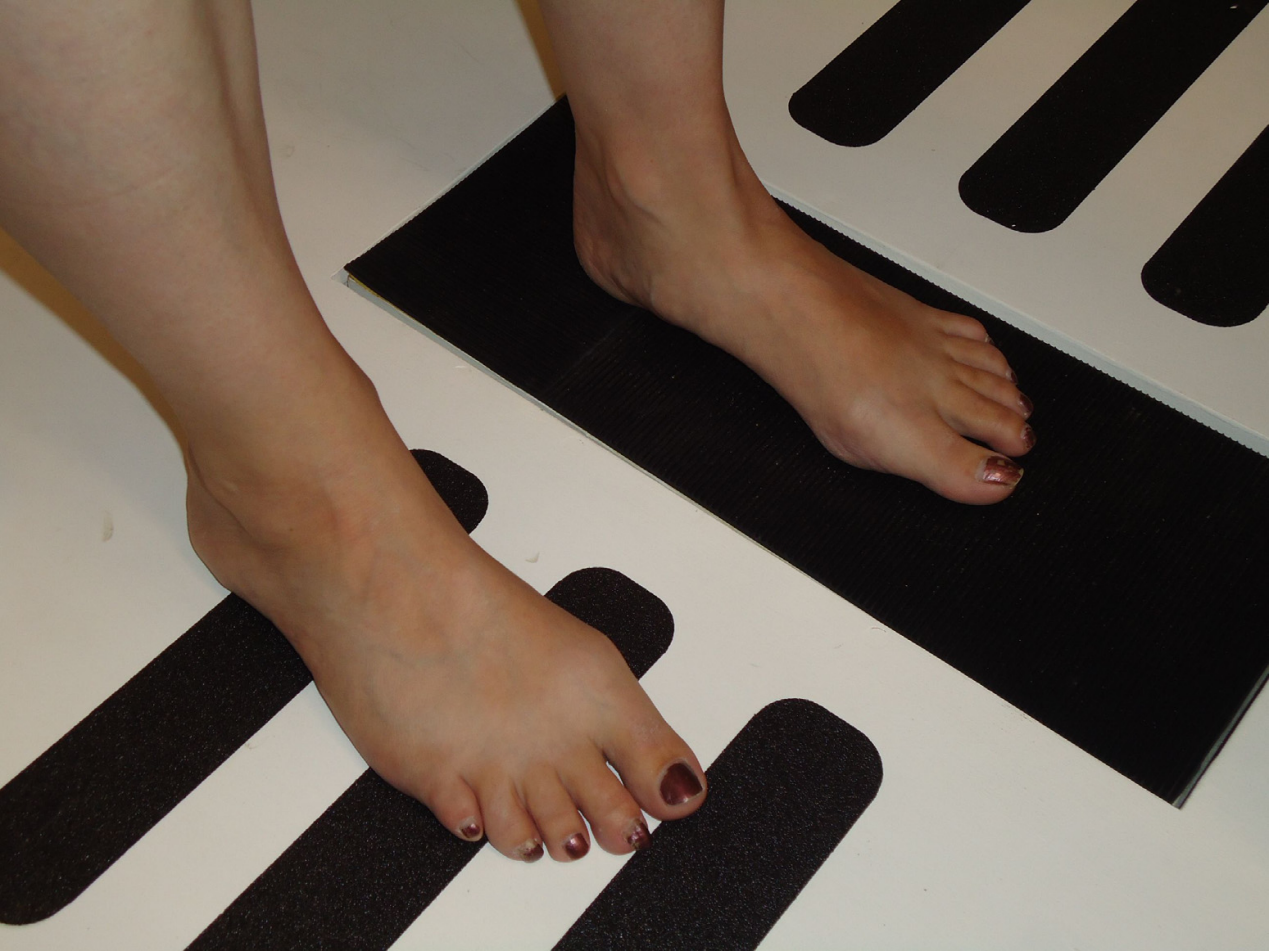
**Positioning on the weight-bearing measurement platform**.

**Figure 2:**
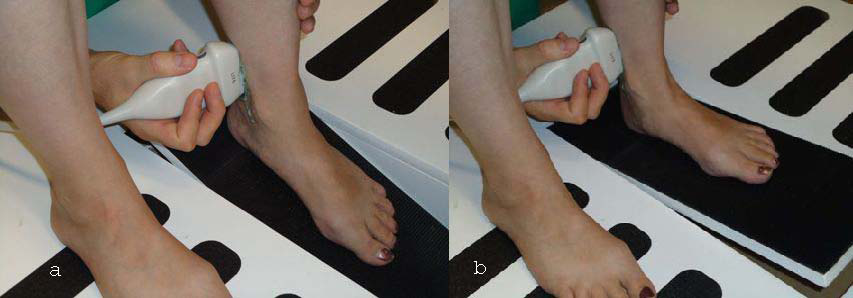
**Weight-bearing position for tibial nerve imaging, (a) ankle in 20° plantarflexion, (b) ankle in 10° dorsiflexion**.

All participants were instructed regarding measurement protocols and practised foot movements on the weight-bearing platform prior to measurement. Each participant was positioned on the weight-bearing measurement platform as demonstrated in Figure 1. The platform was designed to allow one foot to move from 10° plantarflexion to 20° dorsiflexion. The participants left foot was positioned on the platform by the examiner; the medial malleoli was aligned with the pivot point on the platform. The right foot was positioned in a parallel position to the left foot. The tibial nerve was then located and imaged as detailed in the nerve location and measurement procedures. Imaging occurred over a three-second duration as the participant actively moved their left foot from a position of 20° plantarflexion (Figure 2a), to a position of 10° dorsiflexion (Figure 2b).

### Nerve location and measurement procedures

The tibial nerve was located by a transverse scan beginning approximately 1 cm superior to the medial malleolus. The transducer was then moved proximally until the neurovascular bundle was identified. A colour Doppler was used to confirm the location of the tibial artery and assist nerve location, as the tibial artery descends distally with the tibial nerve to enter the tarsal tunnel [22]. The ultrasound transducer was then rotated 90° and aligned longitudinally in the plane of the tibial nerve. The transducer was then moved proximally and distally in the longitudinal plane to ascertain if there was any bifurcation of the tibial nerve and the nerve located was the tibial and not a peripheral branch of the nerve. To confirm the structure located was a nerve, a neural differentiation movement (hip flexion) was performed by the subject. The location was confirmed if the tibial nerve was observed to slide longitudinally under ultrasound imaging. A neural differentiation movement produces excursion in the neural structures in the area rather than moving musculoskeletal structures [3]. The tibial nerve was then imaged with a three-second video loop captured at 30 frames per second, as the ankle moved from a position of 20° plantarflexion to 10° dorsiflexion in the weight-bearing position. Throughout this movement the transducer was held manually by the examiner [MC].

For scanning sessions 1 and 2 three repetitive measurements were taken at one- minute intervals. There was a five- minute interval between scanning session 1 and 2. Between the scanning sessions the transducer was removed, repositioned and the nerve relocated by the examiner.

### Data analysis

The mean (SD) nerve excursion for each session was obtained by averaging three scans within each session.

### Cross-correlation analysis

The video loop of longitudinal nerve excursion captured via ultrasound imaging was converted to digital format (bitmaps). The image size for each of the frames was 800 × 600 pixels. ImageJ (version 1.42, National Institute of Health, Maryland, USA) digitial image analysis software was used to calculate the image resolution and also scale conversion from pixels to millimetres. Each video loop was then analysed offline using a method of frame-by-frame cross-correlation analysis software developed in Mat-lab (MathWorks, Natick, MA, USA) by Dilley et al. [10]. The software determines relative nerve excursion between successive frames in the sequence of ultrasound images. To analyse excursion of the nerve, three rectangular regions-of-interest (ROI) of varied dimensions were selected within the tibial nerve. In the compared frame, the coordinates of the ROI are offset along the horizontal image plane a pixel at a time within a predetermined range [10].

The software compares the grey-scale values from the ROIs between adjacent frames of the image sequence by a correlation coefficient calculation for each individual pixel shift. To reduce shift variability and vertical shifting the compared pixel measurements for the nerve were offset against (subtracted from) pixel shifts measurements within the same ultrasound field, from stationary structures such as subcutaneous layers and bone [10].

### Statistical analysis

All continuous data (age, weight, height, tibial nerve excursion, differences between scanning sessions) were screened for normality through calculation of the Kolmogorov-Smirnov statistic. The mean (SD) was obtained for all continuous data. Intra-rater reliability analysis used Intraclass Correlation Coefficients (ICC, 2,1) and 95% confidence intervals (CI) to quantify reproducibility regarding imaging of the nerve. Reliability findings were interpreted by arbitrary benchmarks initially proposed by Fleiss [23]. The strength of the agreement was poor if the correlation ranged from 0-0.40; fair to moderate if the correlation ranged from 0.40-0.75 and excellent if the correlation ranged from 0.75-1.00.

Standard error of the measurement (SEM) calculations were undertaken to assess the difference between the actual measured score across the images and the estimated "true" scores [24]. The smallest real difference (SRD) was calculated from the SEM and indicates the degree of change that would exceed the trial to trial variability [25]. SRD was calculated by the following formula: SEM × √2 × 2.120 (where 2.120 represents the *t *value of the distribution for a 95% CI (*df *= 15). The SRD percentage was calculated through dividing the SRD by the mean nerve excursion [26]. All tests were calculated using SPSS (V17, SPSS Inc., Chicago, IL).

## Results

The Kolmogorov Smirnov statistic demonstrated normal distributed scores for all continuous data. Descriptive information of tibial nerve excursion, differences between scanning sessions, ICC, SEM and SRD are presented in Table 1. Excellent intra-rater reliability (ICC = 0.93, 95%CI: 0.70-0.96) was found. The degree of measurement error, expressed by the SEM (0.28 mm for session 1 and 0.22 mm for session 2), SRD (0.84 mm for session 1 and, 0.68 mm for session 2) and SRD percentage (27% for session 1 and, 27% for session 2), varied minimally between the scans measured in the weight-bearing position.

**Table 1:** Reliability Indices Determined by the Mean of Three Trials from Two Imaging Sessions

Measurement Position	Session 1 Mean nerve excursion(mm) (± SD)	Session 2 Mean nerve excursion(mm) (± SD)	đ(mm)(± SD)	ICC	ICC 95% CI	SEM (mm)		SRD (SRD%)	
						
						Session 1	Session 2	Session 1	Session 2
**Weight-bearing**	3.03 (1.07)	2.99 (0.86)	0.04 (0.47)	0.93	0.70-0.96	0.28	0.22	0.84 (27)	0.66 (22)

đ = difference (mm) between session 1 & session 2

## Discussion

Our results demonstrated a reduction in tibial nerve excursion (3.00 mm) compared to previous cadaver based studies [8,9]. Coppieters et al. [8] reported a mean longitudinal tibial nerve excursion of 9.5 mm as the ankle was moved from 40° plantarflexion to approximately 15° dorsiflexion during a straight leg raise. The authors reported that excursion was quantified via a digital Vernier calliper with reference points being a suture around the nerve and a fixed marker screwed into cortical bone. Using a similar methodology for measurement of nerve excursion Alshami et al. [9] reported 6.9 mm of longitudinal tibial nerve excursion during a dorsiflexion eversion test, in which the ankle was moved from 0° to 17.1° dorsiflexion, then everted 10°. The major limitation of previous cadevar studies related to the experimental preparation in particular that the Achilles tendon was transected to obtain a physiological range of motion. Therefore, tibial nerve excursion maybe altered by the total ankle range of motion and eversion of the rearfoot as reported in previous studies [8,9].

The differences in nerve excursion may also be attributable to the embalmment process and its potential effects on neural tissue elasticity. Cho et al. [27] speculated that there may be less tissue elasticity in fresh frozen cadavers compared to living tissue. Coppieters and Alshami [28] noted that limited information is known about the effects of freezing and thawing or the impact of embalmment on the mechanical properties of nerves, in particular elasticity. If the embalmment process had a major effect on elasticity and ultimately excursion we would have expected the current results to demonstrate similar levels or more excursion when compared to the cadaver studies. This factor in combination with the limitations of the experimental setup of the cadaver based models may indicate that tibial nerve excursion is influenced in large part by the position of surrounding joints.

The positioning of the knee and hip may have influenced the degree of nerve excursion in the current study. The movements of hip flexion, knee extension and dorsiflexion of the foot increase tension or pre-tension the sciatic and tibial nerve [8,29]. Alshami et al. [29] reported strain in the tibial nerve at the tarsal tunnel is lowest when the positions of the hip or knee do not pretension the sciatic or tibial nerve. Shacklock [3] defined the dynamic relationship between neural tension and neural excursion as the following: in the early part of joint movement the primary event in the nervous system is taking up the slack. In mid-range, the slack is absorbed and the rate of neural sliding increases. Then later, in joint movement, the slack and capacity of nerves to slide has been consumed, causing tension in the nerves to rise. As demonstrated in the current study, mean nerve excursion was lower when measured in the weight-bearing position with the knee extended; preloading the tibial nerve, reducing the capacity of the nerve to slide.

We found longitudinal excursion of the tibial nerve demonstrated excellent intra-rater reliability. Based on a SEM of 0.28 and 0.22 mm for session 1 and 2 respectively, the SRD percentage was calculated and revealed a change in length of greater than 27% (0.84 mm) and 22% (0.67 mm) respectively for session 1 and 2 would be required to be 95% confident that a real change had occurred. Therefore, tibial nerve excursion of greater than 0.84 mm can be considered real change. The ankle range of motion was standardised to a total range of 30° (20° plantarflexion to 10° dorsiflexion) by the measurement platform. Consequently, results displayed low SEM but relatively high SRD values. These findings indicate the benefits of using the measurement platform with the foot in a standardised position to obtain tibial nerve measurements at the ankle for future investigations.

The impact of foot posture was not investigated, which may affect the degree of tibial nerve excursion. Differing foot postures such as a pronated [flatfoot] or supinated [high-arched] foot type may have an influence on the mechanical functioning of the tibial nerve. We can speculate that foot pronation may have a pre-tensioning effect on the nervous system similar to that of the knee extension and hip flexion, potentially reducing the capacity of the tibial nerve to slide longitudinally. A pronated foot type has been associated with increases in pressure in the tarsal tunnel, creating the potential for a compression neuropathy [30]. Specifically the valgus position of the rearfoot associated with a pes planus foot posture is postulated to increase stretch on the tibial nerve, placing increased compressive force on the contents of the tarsal tunnel [31]. Daniels et al. [31] conducted *in-vitro *investigations, concluding that tibial nerve tension was increased in a pes planus foot posture, and postulating that there may be a link to the development of the compressive neuropathy tarsal tunnel syndrome.

As indicated by the SRD there was a relatively high degree of error involved in the measurement of longitudinal nerve excursion. This error maybe explained by the limitations of ultrasound imaging technique employed. The success of ultrasound imaging is operator dependent with the placement of the probe in the exact same position and anatomical plane a potential source of sonographic artefact [32]. The tibial nerve a three-dimensional structure was imaged in a two-dimensional plane, with the nerve essentially representing a thin line at an arbitrary angle in the body. To avoid the effect of anisotropy, the transducer was however kept perpendicular to the nerve throughout the measurement process to avoid creation of this artefact [33].

## Conclusions

In-vivo measurement of longitudinal tibial nerve excursion with frame-by-frame cross-correlation analysis of ultrasound images is a reliable technique, but this technique demonstrated a relatively large measurement error. Future work will include investigating the effect of foot posture on tibial nerve excursion and the assessment of tibial nerve excursion in lower limb conditions such as tarsal tunnel syndrome and plantar fasciitis, where the tibial nerve has been implicated in symptomology. Clinically the technique may also have applications in the assessment of musculoskeletal structures such as tendons allowing for the quantification of excursion in chronic conditions that affect the foot and lower leg such as rheumatoid arthritis and gout and specific tendon pathologies such as posterior tibialis tendon dysfunction.

## Competing interests

The authors declare that they have no competing interests.

## Authors' contributions

MC and KR designed the study. MC and JY collected and input the data. MC and JY conducted the statistical analysis. MC and KR compiled the data and MC, KR and WH drafted the manuscript. All authors read and approved the final manuscript.

## References

[B1] Butler D The Sensitive Nervous System 2000 Adelaide: Noigroup Publications

[B2] Shacklock M Neurodynamics Physiotherapy 1995 81 9 16 10.1016/S0031-9406(05)67024-1

[B3] Shacklock M Clinical Neurodynamics: A new system of musculoskeletal treatment 2005 Edingburgh: Elsevier

[B4] Brown C Gilbert K Brismee J-M Sizer P James C Smith P The effects of neurodynamic mobilization on fluid dispersion within the tibial nerve at the ankle: an unembalmed cadaveric study J Man Manip Ther 2011 19 26 34 10.1179/2042618610Y.0000000003 22294851 PMC3172954

[B5] Driscoll PJ Glasby MA Lawson GM An in vivo study of peripheral nerves in continuity: biomechanical and physiological responses to elongation J Orthop Res 2002 20 370 375 10.1016/S0736-0266(01)00104-8 11918319

[B6] Lundborg G Rydevik B Effects of stretching the tibial nerve of the rabbit. A preliminary study of the intraneural circulation and the barrier function of the perineurium J Bone Joint Surg Br 1973 55 390 401 4707307

[B7] Ogata K Naito M Blood flow of the peripheral nerve effects of dissection, stretching and compression J Hand Surg Br 1986 11 10 14 10.1016/0266-7681(86)90003-3 3958526

[B8] Coppieters MW Alshami AM Babri AS Souvlis T Kippers V Hodges PW Strain and excursion of the sciatic, tibial, and plantar nerves during a modified straight leg raising test J Orthop Res 2006 24 1883 1889 10.1002/jor.20210 16838375

[B9] Alshami A Babri A Souvlis T Coppieters M Biomechanical evaluation of two clinical tests for plantar heel pain: the dorsiflexion-eversion test for tarsal tunnel syndrome and the windlass test for plantar fasciitis Foot Ankle Int 2007 28 499 505 10.3113/FAI.2007.0499 17475147

[B10] Dilley A Greening J Lynn B Leary R Morris V The use of cross-sectional analysis between high-frequency ultrasound images to measure longitudinal median nerve movement Ultrasound Med Biol 2001 27 1211 1218 10.1016/S0301-5629(01)00413-6 11597362

[B11] Dilley A Lynn B Greening J DeLeon N Quantitative in vivo studies of median nerve sliding in response to wrist, elbow, shoulder and neck movements Clin Biomech 2003 18 899 907 10.1016/S0268-0033(03)00176-1 14580833

[B12] Dilley A Lynn B Pang S Pressure and stretch mechanosensitivity of peripheral nerve fibers following local inflammation of the nerve trunk Pain 2005 117 462 472 10.1016/j.pain.2005.08.018 16154692 PMC1402335

[B13] Dilley A Summerhayes C Lynn B An in vivo investigation of ulnar nerve sliding during upper limb movements Clin Biomech 2007 22 774 779 10.1016/j.clinbiomech.2007.04.004 17531363

[B14] Dilley A Odeyinde S Greening J Lynn B Longitudinal sliding of the median nerve in patients with non-specific arm pain Man Ther 2008 13 536 543 10.1016/j.math.2007.07.004 17913563

[B15] Erel E Dilley A Greening J Morris V Cohen B Lynn B Longitudinal sliding of the median nerve in patients with carpal tunnel syndrome J Hand Surg: J Br Soc Sur Hand 2003 28 439 443 10.1016/s0266-7681(03)00107-4 12954253

[B16] Erel E Dilley A Turner S Kumar P Bhatti WA Lees VC Sonographic measurements of longitudinal median nerve sliding in patients following nerve repair Muscle Nerve 2009 41 350 354 10.1002/mus.21501 19813195

[B17] Greening J Dilley A Lynn B In vivo study of nerve movement and mechanosensitivity of the median nerve in whiplash and non-specific arm pain patients Pain 2005 115 248 253 10.1016/j.pain.2005.02.023 15911151

[B18] Echigo A Aoki M Ishiai S Yamaguchi M Nakamura M Sawada Y The Excursion of the Median Nerve during Nerve Gliding Exercise: An Observation with High-resolution Ultrasonography J Hand Ther 2008 21 221 228 10.1197/j.jht.2007.11.001 18652966

[B19] Julius A Lees R Dilley A Lynn B Shoulder posture and median nerve sliding BMC Musculoskelet Disord 2004 5 23 10.1186/1471-2474-5-23 15282032 PMC503391

[B20] Coppieters MW Hough AD Dilley A Different nerve-gliding exercises induce different magnitudes of median nerve longitudinal excursion: an in vivo study using dynamic ultrasound imaging J Orthop Sports Phys Ther 2009 39 164 171 19252262 10.2519/jospt.2009.2913

[B21] Ellis R Hing W Dilley A McNair P Reliability of measuring sciatic and tibial nerve movement with diagnostic ultrasound during a neural mobilisation technique Ultrasound Med Biol 2008 34 1209 1216 10.1016/j.ultrasmedbio.2008.01.003 18343020

[B22] Gray H Standring S Ellis H Berkovitz B Gray's Anatomy: The anatomical basis of clinical practice 2005 39 Edingburgh: Elsevier Churchill Livingstone22431879

[B23] Fleiss J The Design and Analysis of Clinical Experiments 1986 New York: Wiley

[B24] Dudek F The continuing misinterpretation of the standard error of measurement Psychol Bull 1979 86 335 337

[B25] Ota S Ward SR Chen YJ Tsai YJ Powers CM Concurrent criterion-related validity and reliability of a clinical device used to assess lateral patellar displacement J Orthop Sports Phys Ther 2006 36 645 652 17017269 10.2519/jospt.2006.2263

[B26] Campanini I Merlo A Reliability, smallest real difference and concurrent validity of indicies computed from GRF components in gait of stroke patients Gait Posture 2009 30 127 131 10.1016/j.gaitpost.2009.03.011 19428254

[B27] Cho R Braun S Ta K Palesty J Mine R SA S Chang D Thomson J Early passive mobilisation after digital nerve repair and grafting in a fresh cadaver Plast Reconstr Surg 2001 108 386 391 10.1097/00006534-200108000-00017 11496180

[B28] Coppieters MW Alshami AM Coppieters MW Alshami AM Longitudinal excursion and strain in the median nerve during novel nerve gliding exercises for carpal tunnel syndrome J Orthop Res 2007 25 972 980 10.1002/jor.20310 17415752

[B29] Alshami A Babri A Souvlis T Coppieters M Strain in the tibial and plantar nerves with foot and ankle movements and the influence of adjacent joint positions J Appl Biomech 2008 24 368 376 19075306 10.1123/jab.24.4.368

[B30] Barker AR Rosson GD Dellon AL Pressure changes in the medial and lateral plantar and tarsal tunnels related to ankle position: a cadaver study Foot Ankle Int 2007 28 250 254 10.3113/FAI.2007.0250 17296148

[B31] Daniels TR Lau JT Hearn TC The effects of foot position and load on tibial nerve tension Foot Ankle Int 1998 19 73 78 9498578 10.1177/107110079801900204

[B32] Fenster A Downey D Cardinal N Three-dimensional ultrasound imaging Phys Med Biol 2001 46 R67 R99 10.1088/0031-9155/46/5/201 11384074

[B33] Patel S Fessell D Jacobson J Hayes C Holsbeeck M Artifacts, anatomic variants and pitfalls in sonography of the foot and ankle Am J Roentgenol 2002 178 1247 1254 10.2214/ajr.178.5.1781247 11959741

